# Artificial Intelligence Radiotherapy Planning: Automatic Segmentation of Human Organs in CT Images Based on a Modified Convolutional Neural Network

**DOI:** 10.3389/fpubh.2022.813135

**Published:** 2022-04-15

**Authors:** Guosheng Shen, Xiaodong Jin, Chao Sun, Qiang Li

**Affiliations:** ^1^Institute of Modern Physics, Chinese Academy of Sciences, Lanzhou, China; ^2^Key Laboratory of Basic Research on Heavy Ion Radiation Application in Medicine, Lanzhou, China; ^3^Key Laboratory of Heavy Ion Radiation Biology and Medicine of Chinese Academy of Sciences, Lanzhou, China; ^4^University of Chinese Academy of Sciences, Beijing, China

**Keywords:** convolutional neural network (CNN), human organs, CT images, automatic segmentation, Dice similarity coefficient (DSC)

## Abstract

**Objective:**

Precise segmentation of human organs and anatomic structures (especially organs at risk, OARs) is the basis and prerequisite for the treatment planning of radiation therapy. In order to ensure rapid and accurate design of radiotherapy treatment planning, an automatic organ segmentation technique was investigated based on deep learning convolutional neural network.

**Method:**

A deep learning convolutional neural network (CNN) algorithm called BCDU-Net has been modified and developed further by us. Twenty two thousand CT images and the corresponding organ contours of 17 types delineated manually by experienced physicians from 329 patients were used to train and validate the algorithm. The CT images randomly selected were employed to test the modified BCDU-Net algorithm. The weight parameters of the algorithm model were acquired from the training of the convolutional neural network.

**Result:**

The average Dice similarity coefficient (DSC) of the automatic segmentation and manual segmentation of the human organs of 17 types reached 0.8376, and the best coefficient reached up to 0.9676. It took 1.5–2 s and about 1 h to automatically segment the contours of an organ in an image of the CT dataset for a patient and the 17 organs for the CT dataset with the method developed by us, respectively.

**Conclusion:**

The modified deep neural network algorithm could be used to automatically segment human organs of 17 types quickly and accurately. The accuracy and speed of the method meet the requirements of its application in radiotherapy.

## Introduction

Radiation therapy, which deliver lethal doses to a target volume while sparing the surrounding normal tissues as much as possible, has been a key modality of cancer treatments. So, accurate and rapid identification and delineation of normal organs and target volumes are the basis for precision radiotherapy (1–5). In the conventional workflow of radiation therapy, medical doctors spend too much time dealing with CT images manually and the accuracy of organs segmentation depends heavily on the professional skills of medical doctors (6). An amateurish job of human organ contour segmentation could seriously influence the curative effect of radiotherapy.

The common organ segmentation algorithms with conventional automaticity and semi-automaticity are based on gray value, texture, template setting, and other features of CT images. Therefore, these methods are often apt to failure in identifying all organs in CT images and delineating the contours (7–9).

With the rapid development of artificial intelligence (AI) technologies, especially in the application of fundamental convolutional neural networks (CNN), the medical image recognition and segmentation are getting more and more higher reliability and accuracy, and this is also a hot issue in current research (10–15), a lot of new CNN net (such as BDR-CNN-GCN) were designed to classified the cancer and achiever a significantly effective (16). The appearance of semantic-based full convolutional network and U-Net enables AI technology to achieve pixel-to-pixel prediction, which had a wide impact on the field of computer vision as soon as they appeared. It has been widely used in the image segmentation, object detection, object recognition, and so on. In the field of biomedical images, CNN is widely used in automatic detection and classification of diseases, prediction of therapeutic effects, segmentation and recognition of special tissues and organs, etc. (15, 17–21). The medical data which has been annotated by experienced physicians was used to train and validate the CNN net, so the CNN can be used to predict and extracts the features from new medical data. Based on these features which obtained from the designed CNNs, the algorithm can accurately predict and segment the medical images (22–26).

In order to achieve the precision radiotherapy, automatic and accurate identification, and segmentation of human organs in medical images are absolutely necessary. Therefore, we designed and modified a U network of CNNs (BCDU-Net: Bi-Directional ConvLSTM U-Net with Dense Connected Convolutions) (27–29), and CT images and corresponding organs (RT-structure) data set from 339 patients were applied to train, validate, and test the network in our work. The method of automatic and accurate segmentation of human organs in medical image definitely can provide support for decreasing the workload of physicians and the development of precision radiotherapy in the future.

## Materials and Methods

### Test Data

In this study, the data were randomly selected from more than 22,000 CT images and corresponding tissue and organ contours from 339 patients who received radiotherapy in a tumor hospital in 2018. All of these tissue and organ contours which had been used in radiotherapy were generated and verified by several experienced physicians using the conventional commercial treatment planning system in the hospital.

The CT images and corresponding tissue and organ contours used in the experiment are outlined as follows: 984 bladder images, 451 brainstem images, 451 left eye (eye-L) images, 359 right eye (eye-R) images, 1,778 left femur (femur-L) images, 1,603 right femur (femur-R) images, 2,059 heart images, 699 intestine images, 964 left kidney images, 908 right kidney images, 2,890 liver images, 1,491 left lung images, 3,397 right lung images, and 754 mandible images, 1,673 rectum images, 550 spleen images, and 890 stomach images.

In this study, 70% of the images of each organ contours and corresponding CT images were randomly selected for training, 20% were selected for verification and 10% were selected for testing.

### Deep CNN Algorithm

In order to realize automatic segmentation of organs with CT images, a new U-Net algorithm based on the deep CNN algorithm was designed and developed using python language, in which a BCDU-Net network algorithm of deep neural network which has been published by Azad et al. (27) was referred and improved by us in the present work. In the BCDU-net algorithm the authors included BN after each up-convolutional layer to speed up the network learning process. And BN can help the network to improve the performance. Also the network with dense connections could improve the accuracy. The key idea of dense convolutions is sharing feature maps between blocks through direct connection between convolutional block. Consequently, each dense block receives all preceding layers as input, and therefore, produces more diversified and richer features. So the BCDU-net has a better preference (28, 29).

**Figure 1:**
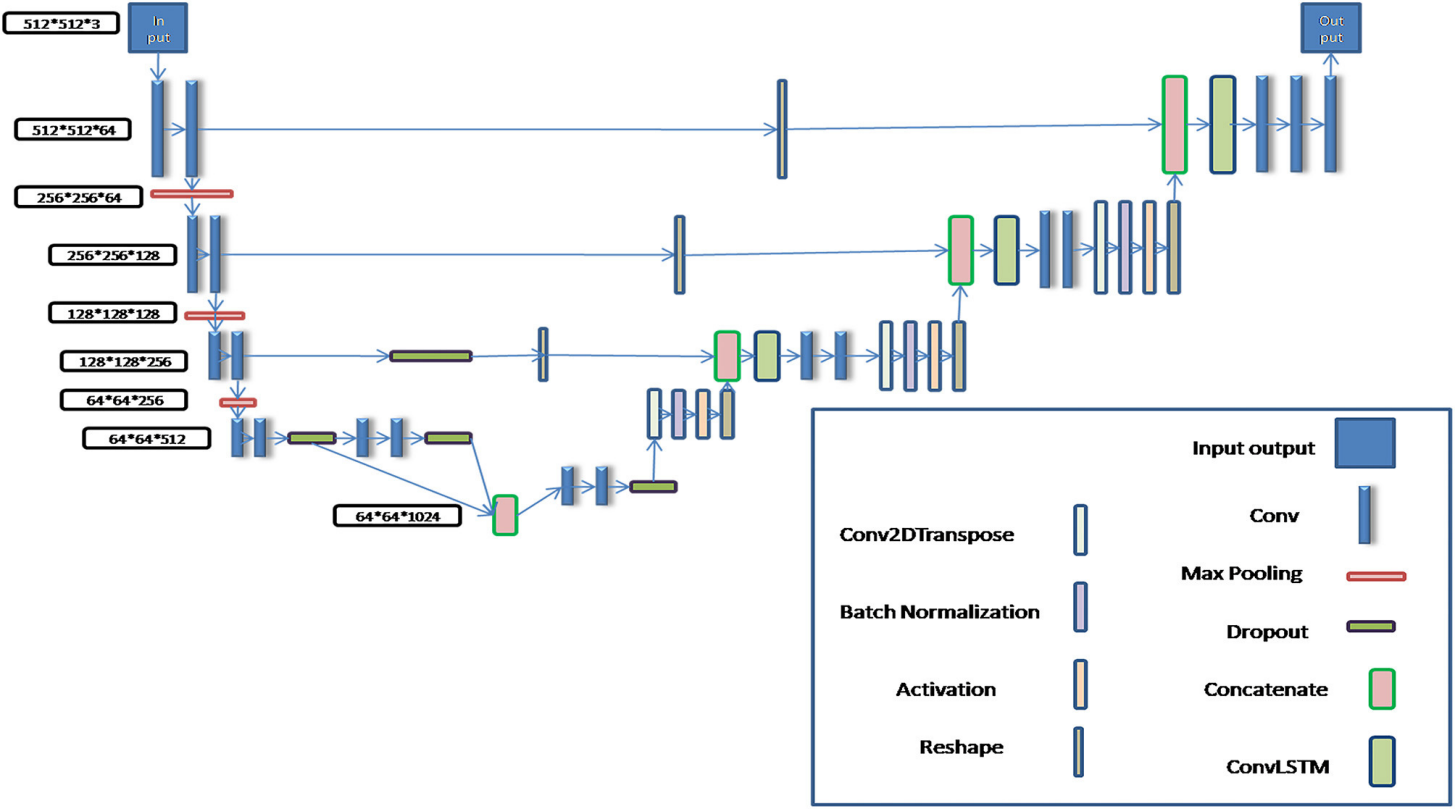
The schematic diagram of the modified BCDU-Net algorithm.

The schematic diagram of the modified algorithm is shown in Figure 1. In the conventional U-Net algorithm, input images are directly copied and added into the deconvolution decoder part from the code part of network, so that the automatic segmentation prediction could be realized. The Bi-Directional ConvLSTM algorithm (28, 29) is used to extract features in the BCDU-Net network compared with the conventional U-Net one. In this work, several conventional U-Net network algorithms were tested and the BCDU-Net model showed an excellent performance in automatic organ recognition with CT images. Therefore, the BCDU-Net model algorithm was modified for use in this work.

In the process of algorithm development, in order to make the BCDU-Net network model suitable for the input of 512^*^512^*^3 CT images and the output of 512^*^512^*^3 tissue and organ contour sets, it was modified by adding two convolution computations behind the deconvolution computing layer so as to obtain multi-channel segmentation images. Then, the predicted contours of organs were automatically output.

### Assessment Method

Accuracy, Precision, and Dice similarity coefficient (DSC) were used to evaluate the algorithm effectiveness in this paper.

Recall: the proportion of correct prediction results in all test data. The threshold range of accuracy is [0,1], the larger the value of accuracy is, the better the results are.

Precision: the proportion of correct prediction. The threshold range of precision is [0,1], the larger the value of accuracy is, the better the results are.

DSC: an important parameter to evaluate the effect of network prediction. Its calculation formula is as follows:


(1) DSC=2 ∗ (Precison ∗ Recall)/(Precison+Recall)

The threshold range of DSC is [0,1], the closer it is to 1, the more accurate the prediction results will be.

In this paper, all of the mentioned evaluation parameters were used to evaluate the prediction results of each organ contours.

### Testing Platform

The hardware platform used in this work is as follows: Dell T5820/P5820X (tower workstation); CPU: I7-7800x 6-core 3.5 ghz Core X series; Graphics GPU: Nvidia Titan RTX-24G; Memory: DDR4 32 GB; Hard disk: solid state 1T+ mechanical 4T.

Operating system: Ubuntu Linux 16.04; Development tools: Spyder + Tensorflow + Keras; Development language: Python.

## Results

More than 22,000 CT images and corresponded organ contours from 329 patients were randomly extracted, in which 70% was for training set, 20% for validation set and 10% for testing set. The labeled CT images and corresponded organ contour images were used to train and validate the algorithm modified in this paper. And then the weight parameters of the modified BCDU-Net algorithm model were obtained.

According to the acquired model parameters mentioned above, the testing set was calculated and examined. The performance of the modified algorithm for automatic organ segmentation in CT images is shown in Table 1. The organ contours segmented automatically by the algorithm were similar to those delineated by physicians manually. The model parameters including DSC, Accuracy, Recall, and Precision evaluation ones were served to evaluate the segmentation effectiveness of each organ in the validation and testing sets. In our work, the BCDU-Net CNN algorithm model was used to automatically segment different organs with the different training parameters such as epoch learning rate. The CT images which were randomly selected from the patients were put into the network model for training, and then the contours of different organs which were delineated automatically by the AI technology and manually by medical doctors were evaluated with the similarity coefficients, respectively. The results are given in Table 2. Most of the DSC values were better than 0.85 and among them the best even reached up to 0.9676. Generally, the automatic segmentation results met the requirements of clinical practice.

**Table 1:** The result of manual and automatic organ segmentation.

	**Input CT image**	**Organs with manual segmentation**	**Organ with automatic segmentation**
Bladder	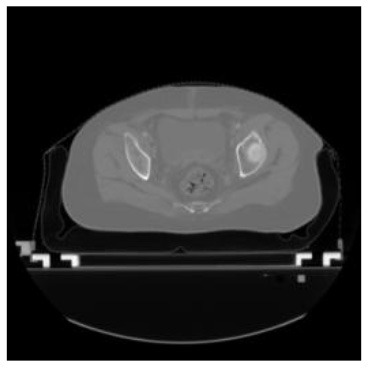	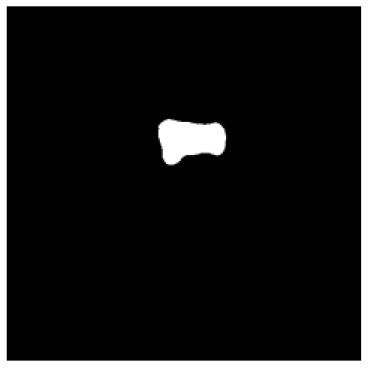	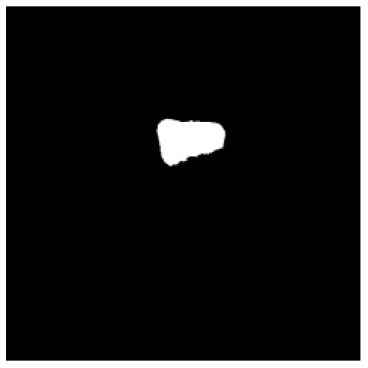
Brainstem	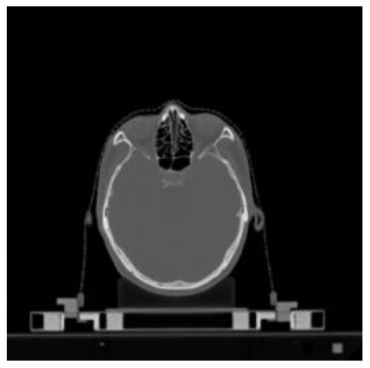	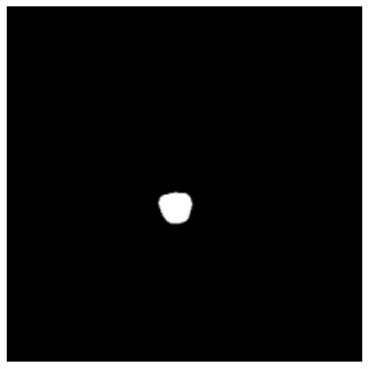	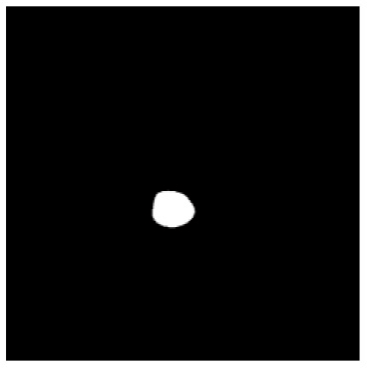
Eye-L	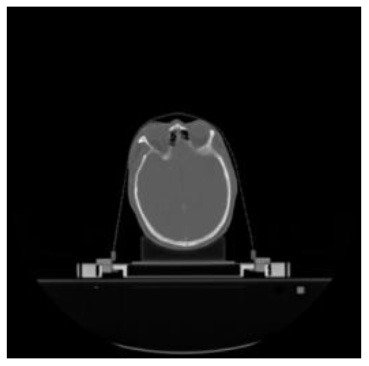	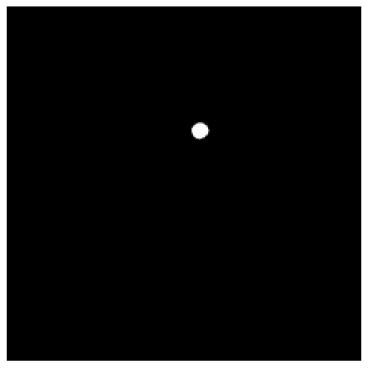	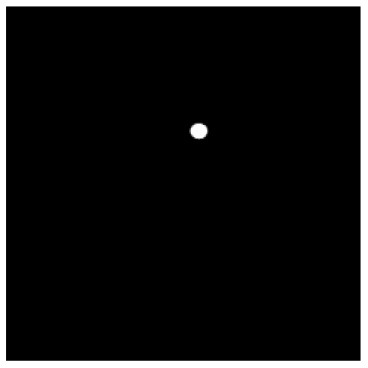
Eye-R	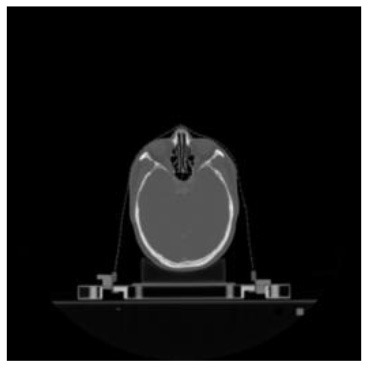	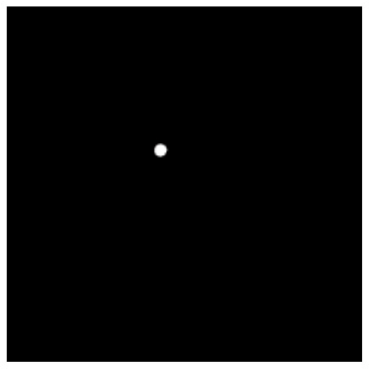	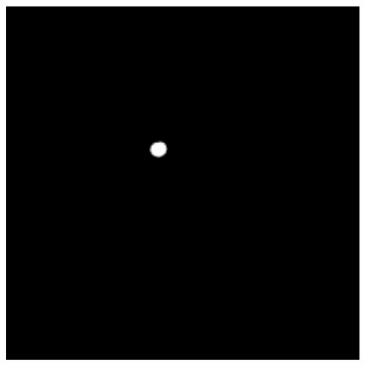
Femur-L	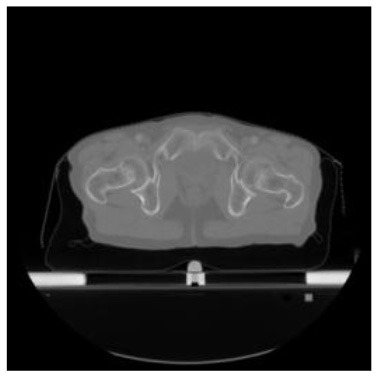	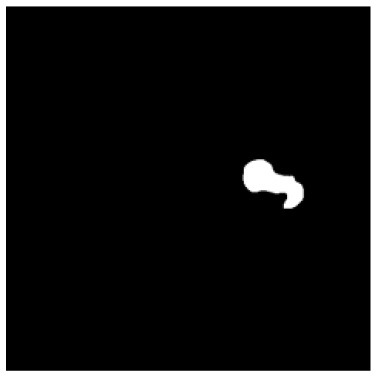	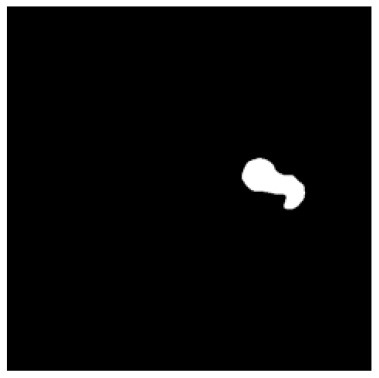
Femur-R	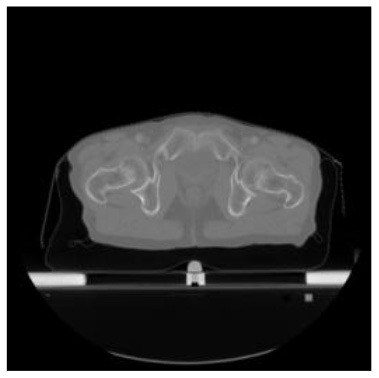	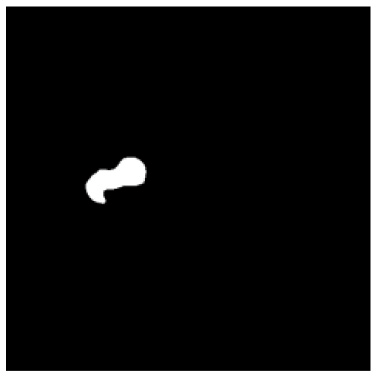	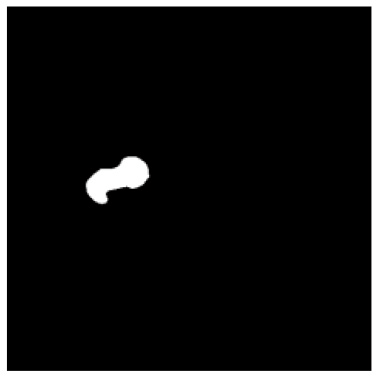
Heart	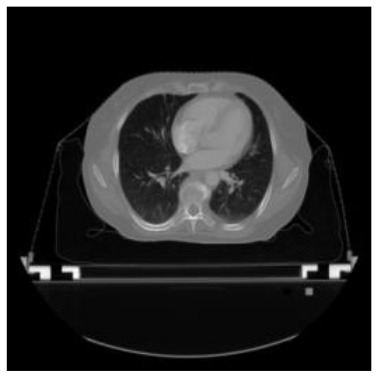	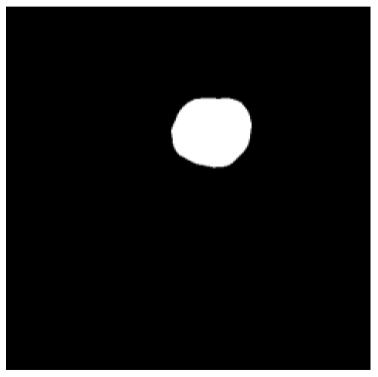	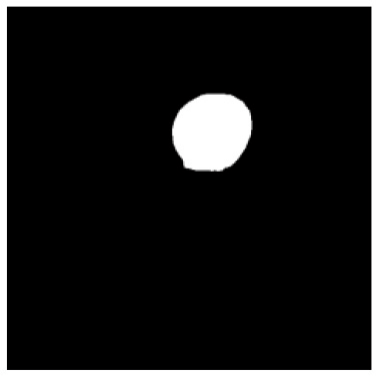
Intestine	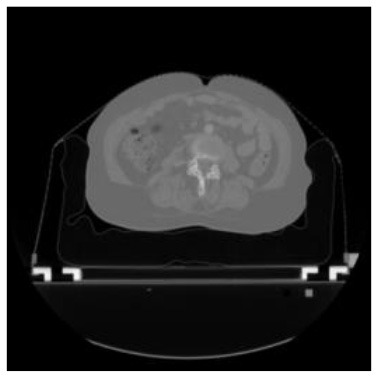	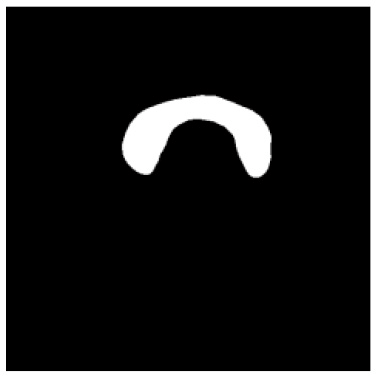	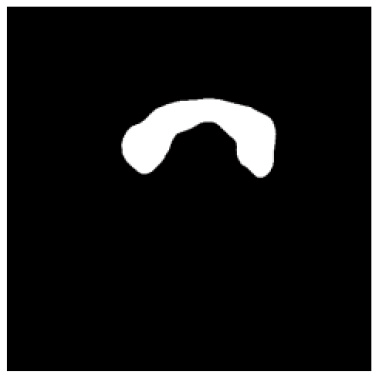
Kidney-L	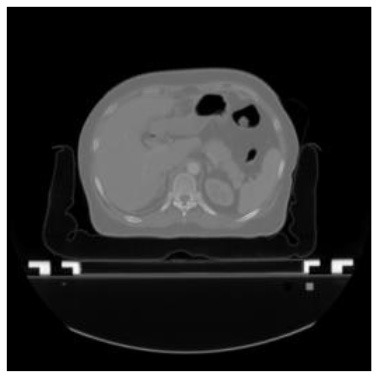	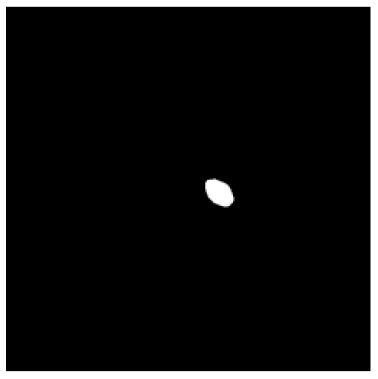	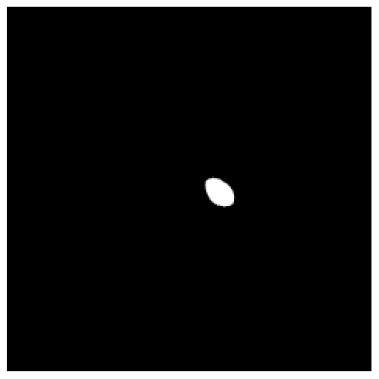
Kidney-R	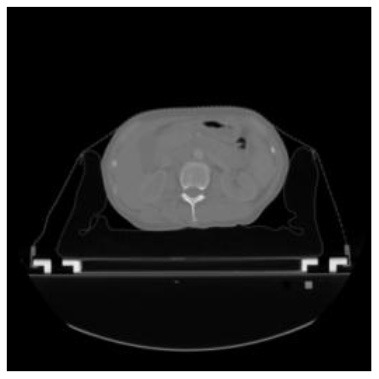	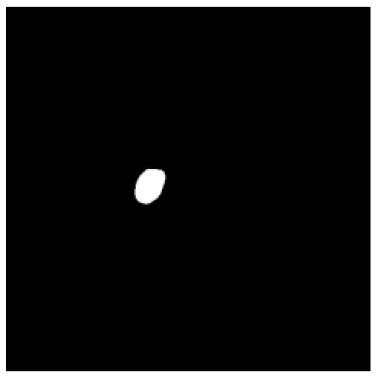	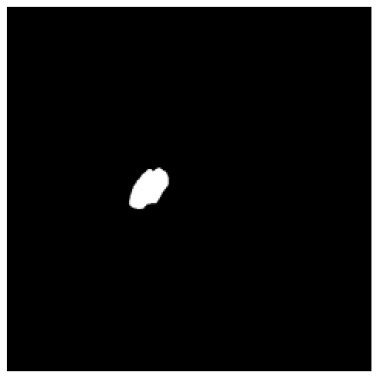
Liver	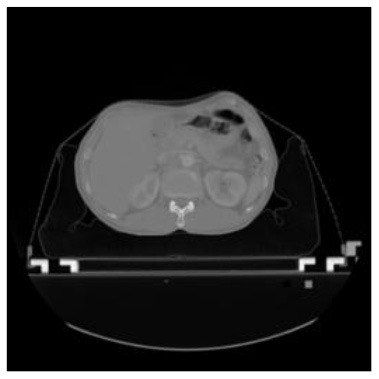	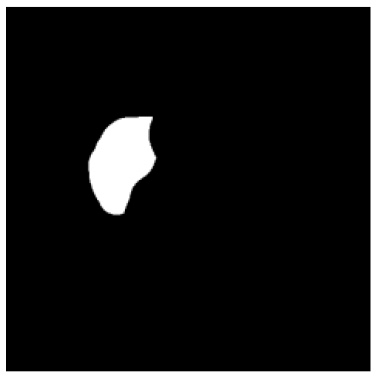	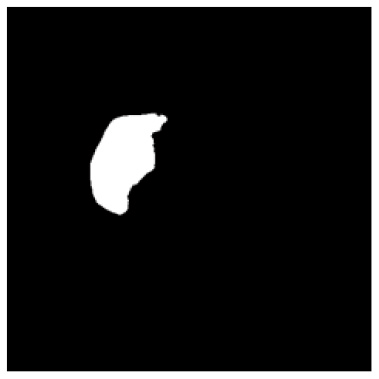
Lung-L	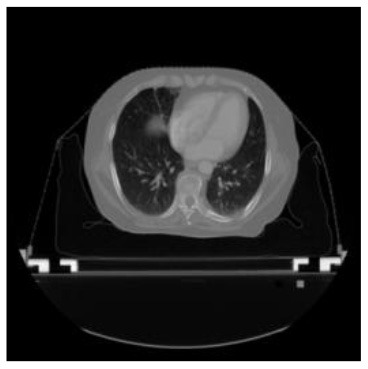	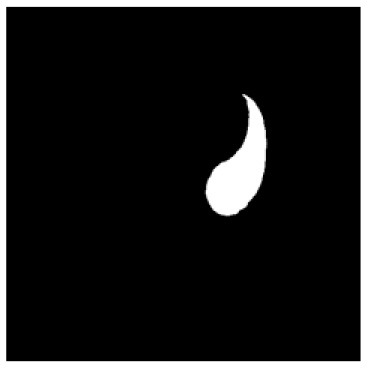	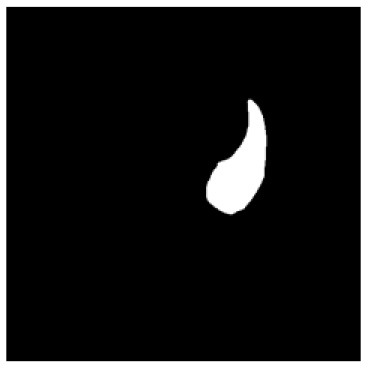
Lung-R	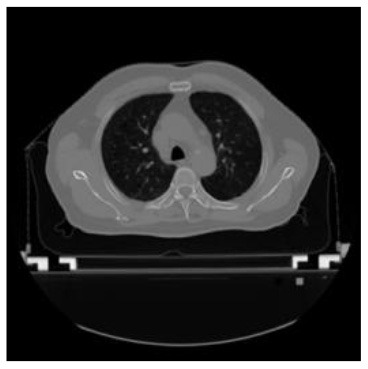	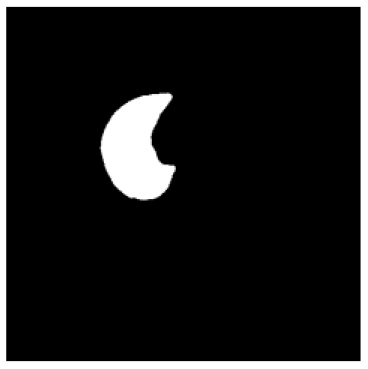	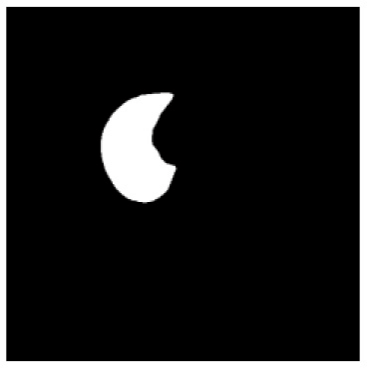
Mandible	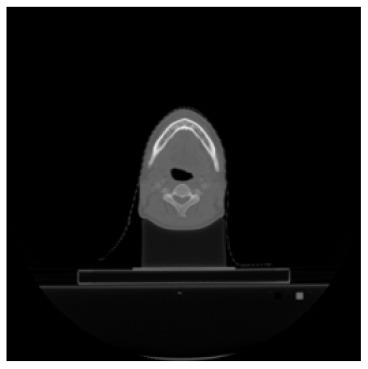	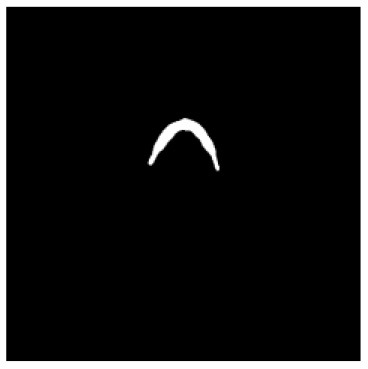	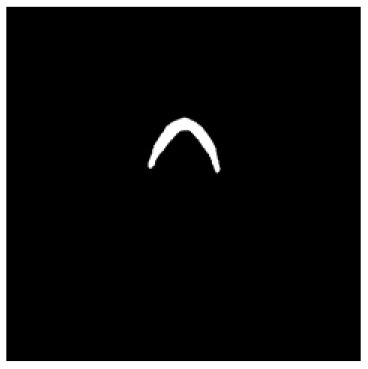
Rectum	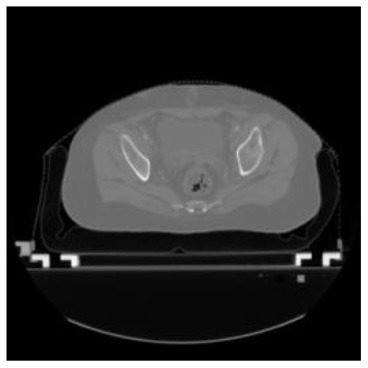	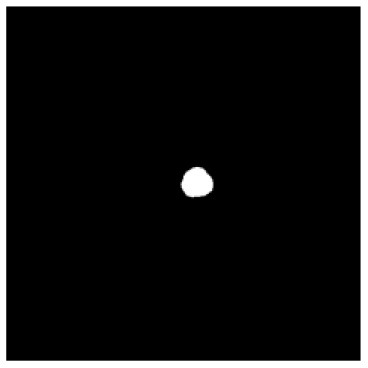	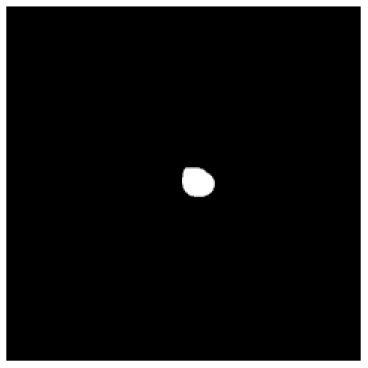
Spleen	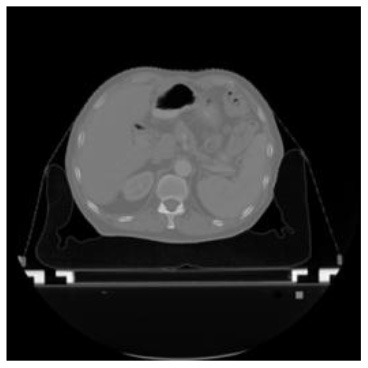	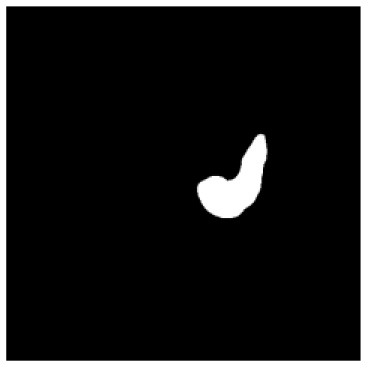	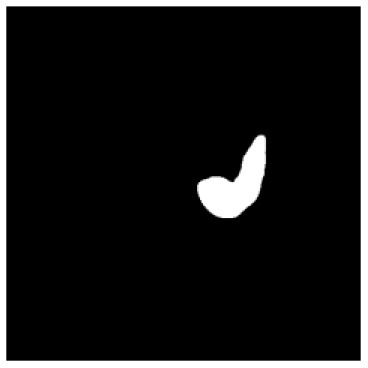
Stomach	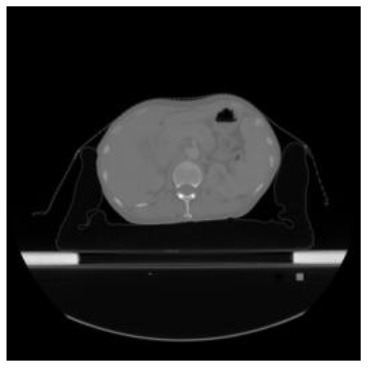	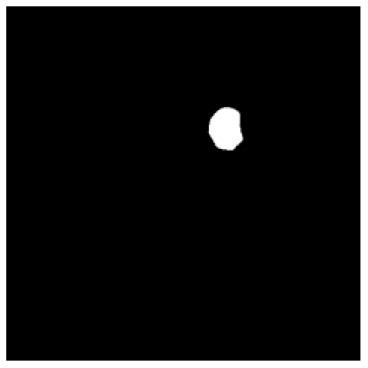	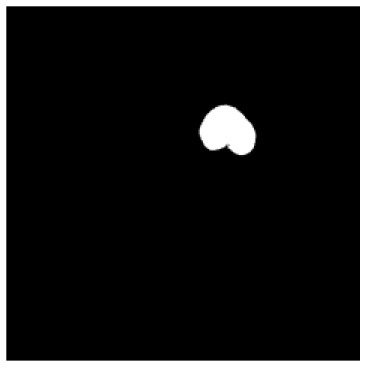

**Table 2:** Evaluation for the automatic organ segmentation.

	**DSC**	**Accuracy**	**Recall**	**Precision**
Bladder	0.8403	0.9977	0.6826	0.9981
Brainstem	0.6786	0.9987	0.6934	0.9925
Eye-L	0.8839	0.9996	0.9537	0.9997
Eye-R	0.8690	0.9995	0.9147	0.9997
Femur-L	0.9357	0.9991	0.9668	0.9993
Femur-R	0.9405	0.9991	0.9586	0.9994
Heart	0.9086	0.9948	0.9727	0.9954
Intestine	0.5084	0.9745	0.8340	0.9767
Kidney-L	0.9313	0.9992	0.9650	0.9994
Kidney-R	0.8822	0.9987	0.9728	0.9988
Liver	0.9221	0.9948	0.9071	0.9979
Lung-L	0.8879	0.9960	0.8434	0.9989
Lung-R	0.9676	0.9977	0.9741	0.9986
Mandible	0.8252	0.9982	0.8976	0.9987
Rectum	0.6782	0.9981	0.5585	0.9997
Spleen	0.9082	0.9978	0.9087	0.9989
Stomach	0.6717	0.9950	0.5289	0.6563

## Discussion

At present, it takes medical doctors a lot of time and energy to identify and delineate human organs in CT images for radiotherapy treatment planning. High accuracy and efficiency of manual segmentation is always a big challenge for medical doctors. With the development of AI technology, the performance of the deep learning CNN algorithm which is used in image processing becomes better, and the CNN algorithm gets more applications in medical images processing for automatic detection of diseases and delineation of specific tissues and organs (malignant and benign). New BDR-CNN-GCN algorithm had been designed and used to classify breast cancer and achieved a better results (16). Also some new AI algorithms were designed and applied in different aspects of healthcare which strongly support the development of healthcare automation technology (2–4, 7–9). In our work, the BCDU-Net deep learning CNN model was modified and used for training and validation via 22,000 CT images and corresponding human organs 17 type from 339 patients. Compared with the manual segmentation, the average DSC value of the modified algorithm for the automatic segmentation of 17 type human organs was 0.8376, and the best DSC coefficient was up to 0.9676. Moreover, the DSC coefficient of 13 in 17 organs was better than 0.82. Obviously, the effectiveness of the algorithm after modification was improved.

The number of the images for each human organ in the 17 type, which were employed for training and validation, was different. So various epoch values were set when we trained for the different organs. The results are given in Table 3. The epoch value was set small when the number of the training images was large. Conversely, the epoch value was set large when the number of the training images was small. So, the accuracy of the automatic organ segmentation was improved, and the DSC value became better.

**Table 3:** The training epoch and CT image numbers for the different organs.

**Organ**	**Number of images**	**Epoch**
Bladder	1,467	80
Brainstem	984	100
Eye-L	451	120
Eye-R	359	120
Femur-L	1,778	60
Femur-R	1,603	60
Heart	2,059	60
Intestine	699	100
Kidney-L	964	100
Kidney-R	908	100
Liver	2,890	50
Lung-L	1,491	80
Lung-R	3,397	40
Mandible	754	100
Rectum	1,673	60
Spleen	550	120
Stomach	890	100

In this work, the DSC values of four organs (intestine, stomach, rectum, and brainstem) were lower than those of the other organs. The reason probably was that it was difficult to split these four organs accurately by medical doctors. So, the labeling quality of the images which were used to train and validate the network was poor. It took 6–8 h to train and validate the modified BCDU-Net algorithm model parameters for each organ. However, the contour of an organ could be segmented automatically in about 1.5–2 s from CT image. Clearly, the automatic organ segmentation with the modified algorithm is much higher efficient than manual delineation by medical doctors.

Even so, we will improve the precision of the modified model through cooperating closely with more experienced medical doctors, making the proposed method to be applied in clinical practice as early as possible.

## Conclusion

To achieve accurate automatic organ segmentation in CT images, the structure of the BCDU-Net CNN algorithm model was designed and improved. More than 22,000 CT images and the contours of human organs of 17 types from 339 patients were applied to train and validate the CNN algorithm model. So, the parameters of the algorithm model were obtained. The performance of the algorithm with an average DSC coefficient of 0.8376 and time consumption of about 1.5–2 s was obtained. Thus, the algorithm could be used to segment human organs of 17 types in CT images automatically and efficiently. More cooperation with experienced medical doctors definitely makes the modified model more suitable for clinical use.

## Data Availability Statement

The datasets presented in this study can be found in online repositories. The names of the repository/repositories and accession number(s) can be found at: https://pan.cstcloud.cn/s/gO5MpMG9TNQ.

## Ethics Statement

The studies involving human participants were reviewed and approved by the Academic Committee of the Institute of Modern Physics, Chinese Academy of Sciences. The patients/participants provided their written informed consent to participate in this study.

## Author Contributions

GS and QL: conception, design, and experimental testing. XJ and CS: administrative support and data interpretation. All authors contributed to the article and approved the submitted version.

## Funding

This work was jointly supported by the Key Deployment Project of Chinese Academy of Sciences (Grant No. KFZD-SW-222) and the West Light Foundation of Chinese Academy of Sciences [Grant No. (2019)90].

## Conflict of Interest

The authors declare that the research was conducted in the absence of any commercial or financial relationships that could be construed as a potential conflict of interest.

## Publisher's Note

All claims expressed in this article are solely those of the authors and do not necessarily represent those of their affiliated organizations, or those of the publisher, the editors and the reviewers. Any product that may be evaluated in this article, or claim that may be made by its manufacturer, is not guaranteed or endorsed by the publisher.
